# KRAS Subtype Modifies Outcomes with Immunomodulatory Therapy in Advanced Pancreatic Ductal Adenocarcinoma: Evidence from Early-Phase Trials

**DOI:** 10.3390/cancers18132037

**Published:** 2026-06-23

**Authors:** Dilsa Mizrak Kaya, Yangruijue Ma, Tarik Demir, Nicole J. Altomare, Aparna Kalyan, Sheetal Kircher, Mary Mulcahy, Al B. Benson, Ruohui Chen, Devalingam Mahalingam

**Affiliations:** 1 Department of Developmental Therapeutics, Robert H. Lurie Comprehensive Cancer Center, Division of Hematology/Oncology, Feinberg School of Medicine, Northwestern University, Chicago, IL 60611, USA; dmizrak@tobbetuhastanesi.com.tr (D.M.K.);; 2 Department of Medical Oncology, TOBB University of Economics and Technology, 06560 Yenimahalle, Ankara, Türkiye; 3 Department of Preventive Medicine, Division of Biostatistics and Informatics, Feinberg School of Medicine, Northwestern University, Chicago, IL 60611, USA; anna.ma@northwestern.edu (Y.M.);; 4 Department of Internal Medicine, Division of Medical Oncology, The Ohio State University Comprehensive Cancer Center, Columbus, OH 43210, USA; 5 Department of Medicine, Division of Hematology/Oncology, Feinberg School of Medicine, Northwestern University, Chicago, IL 60611, USAalbenson@nm.org (A.B.B.III)

**Keywords:** early phase, immunomodulator therapy, KRAS subtype, pancreatic cancer, survival

## Abstract

**Background:** Pancreatic ductal adenocarcinoma (PDAC) is characterized by a near-universal presence of *KRAS* mutations and limited responsiveness to immunotherapy. Biologic heterogeneity among *KRAS* subtypes may shape tumor immunobiology and treatment resistance. **Methods:** We evaluated 109 patients with advanced PDAC treated in early-phase trials between August 2014 and August 2023. Overall survival (OS) and progression-free survival (PFS) were estimated using the Kaplan–Meier method, and Cox proportional hazard models were used to assess the clinical and molecular predictors of survival. **Results:** Among the 109 patients, 64% harbored *KRAS* mutations, 18% were *KRAS* wild type, and 17% had an unknown *KRAS* status. The median age was 65 years, and 83% had liver metastases. Among the *KRAS*-mutant tumors, subtype distribution was G12D (46%), G12V (31%), G12R (13%), and other variants (10%). Immunomodulatory agents were administered to 39% of the patients, most commonly in the first-line setting (49%). The median OS and PFS for the entire cohort were 5.65 and 2.73 months, respectively. The restricted mean OS (7.54 vs. 8.65, *p* = 0.53) and PFS (3.82 vs. 4.24, *p* = 0.70) did not differ between patients with *KRAS* mutants and wild-type *KRAS*. Among patients with *KRAS* mutations, receipt of immunomodulatory therapy was associated with shorter OS compared with those who did not receive immunomodulatory therapy, with the strongest association observed in the *KRAS* G12D subgroup. This pattern was not observed in *KRAS* wild-type tumors. On univariate analysis, immunomodulatory therapy exposure, number of prior treatment lines, and presence of liver metastases were each associated with inferior OS. On multivariable analysis, immunotherapy exposure demonstrated a non-significant trend toward inferior OS (hazard ratio [HR] 1.61, 95% Cl 0.98–2.66; *p* = 0.06), while the other variables remained independently associated with worse OS, suggesting confounding in the unadjusted association between immunomodulatory therapy and survival. **Conclusions:** Among patients with *KRAS*-mutant PDAC—particularly those with G12D—receipt of immunomodulatory therapy in early-phase trials was associated with shorter OS in this exploratory analysis. These findings should be considered hypothesis-generating and require validation in prospective, *KRAS*-subtype-informed studies.

## Simple Summary

Pancreatic cancer remains one of the most difficult cancers to treat, and most patients experience limited benefit from the currently available therapies. Mutations in the *KRAS* gene are common in pancreatic cancer, but it is unclear whether different *KRAS* subtypes influence the outcomes of emerging immunomodulatory treatments. In this study, we evaluated patients with advanced pancreatic cancer enrolled in early-phase clinical trials and examined the relationship between *KRAS* status, *KRAS* subtype, and treatment outcomes. We found that overall outcomes were similar between patients with and without *KRAS* mutations, but exploratory analyses suggested that specific *KRAS* subtypes respond differently to immunomodulatory approaches. These findings highlight the importance of considering the *KRAS* subtype when designing future clinical trials and developing personalized treatment strategies for pancreatic cancer.

## 1. Introduction

Pancreatic ductal adenocarcinoma (PDAC) is the third leading cause of cancer-related mortality in the United States [1]. The incidence of PDAC has steadily increased among both males and females since 2001 [2]. Survival outcomes have modestly improved among patients diagnosed with localized disease; however, this subgroup represents only 15–20% of all PDAC cases [3]. Given the rising incidence and persistently poor prognosis of this highly lethal malignancy, there is an urgent need to develop more effective therapeutic strategies for PDAC.

Over the past two decades, three combination chemotherapy regimens have been established as preferred first-line treatments for patients with advanced PDAC: FOLFIRINOX (5-fluorouracil, leucovorin, irinotecan and oxaliplatin) [4], gemcitabine + albumin-bound paclitaxel [5], and NALIFIROX (5-fluorouracil, leucovorin, liposomal irinotecan and oxaliplatin) [6]. Despite these advances, there are no well-established standard treatment options beyond the first line. Second-line therapy typically involves switching to an alternative chemotherapy backbone and is guided by patient performance status, the presence of actionable molecular alterations, and the availability of clinical trials [7].

Mutations in v-Kiras2 Kirsten rat sarcoma viral oncogene homolog (*KRAS*) are detected in more than 90% of pancreatic cancer tissue samples [8]. Pancreatic intraepithelial neoplasms (PanINs), the most common precursors lesions of PDAC, harbor *KRAS* mutations in approximately 96% of cases, supporting *KRAS* activation as a key initiating event in pancreatic carcinogenesis [9]. The immune microenvironment surrounding PanINs is characterized by inflammatory yet immunosuppressive stromal components, which may enable precursor lesions to evade immune surveillance [10]. Additional genetic alterations, including loss-of-function mutations in *TP53* (64%), *SMAD4* (21%), and *CDKN2A* (17%), typically occur at later stages of disease progression [11,12,13,14].

The distribution of *KRAS* mutation subtypes varies across cancer types. In PDAC, the majority of *KRAS* mutations occur at codon 12, with reported prevalence in descending order of G12D (35–40%), G12V (20–35%), G12R (10–20%), Q61H (5–10%), G12C (1–2%), and other rare variants [8,15,16]. Emerging evidence suggests that specific *KRAS* mutation subtypes are associated with distinct biological behaviors and clinical outcomes [11,16,17,18]. Prior studies have demonstrated that patients with PDAC with *KRAS* G12D and *KRAS* Q61 mutations experience shorter median overall survival (OS) compared with those harboring *KRAS* G12R mutations or *KRAS* wild-type tumors [16]. However, the biological mechanisms underlying these prognostic differences remain incompletely understood. Until recently, *KRAS* was widely considered an undruggable target, further limiting therapeutic exploitation of these molecular distinctions [19,20,21].

The clinical efficacy of immunomodulatory therapies in PDAC has been limited. This resistance has been attributed to multiple factors, including low tumor antigenicity, a profoundly immunosuppressive tumor microenvironment (TME), dense desmoplastic stroma [22], and the modest activity of currently available immunotherapeutic agents [23]. *KRAS* mutations contribute significantly to this hostile immune milieu by promoting cytokine secretion, recruitment of myeloid-derived suppressor cells, and suppression of CD8+ T-cell activity [24,25,26]. Downregulation of *KRAS* G12D DNA was associated with an increase in intratumoral CD8+ T cells [27,28]. A more immunosuppressive TME has also been described in *KRAS* G12D-mutant lung cancer [29,30]. Preclinical studies in PDAC models have shown that inhibition of *KRAS* G12D can partially reverse immune suppression, providing a strong biological rationale for investigating *KRAS*-dependent immunotherapy responses [31,32].

Immunomodulatory therapies encompass a broad range of strategies, including immune checkpoint inhibitors, adoptive cellular therapies, cancer vaccines, cytokine-based treatments, immune agonists, oncolytic viruses, antibody–drug conjugates, and small-molecule immune modulators, all aimed at enhancing or restoring antitumor immune responses.

In this study, we aimed to evaluate whether *KRAS* mutation subtypes influence outcomes following immunomodulatory therapy in patients with advanced PDAC. We also assessed the impact of additional clinical and molecular variables on treatment outcomes. To our knowledge, this is the first study to systematically evaluate immunomodulatory therapy outcomes across distinct *KRAS* subtypes in advanced PDAC. These findings will support the development of *KRAS*-subtype-guided combination strategies incorporating immunomodulatory therapies to improve outcomes in this challenging disease.

## 2. Patients and Methods

### 2.1. Study Design and Population

We conducted a retrospective cohort study of 109 patients with advanced PDAC treated in early-phase clinical trials between August 2014 and August 2023 at the Department of Developmental Therapeutics, Northwestern University Lurie Cancer Center. Patients were identified from a prospectively maintained, institutionally approved database. This study was conducted in accordance with the Declaration of Helsinki and Good Clinical Practice guidelines, with approval from the institutional review board.

Clinical variables collected included age at trial enrollment, sex, prior radiation therapy to the primary tumor, history of pancreatic resection, number of prior systemic therapy lines before early-phase trial enrollment, presence of liver metastases at trial entry, exposure to immunomodulatory therapy within the trial, and *TP53*, *SMAD4*, *CDKN2A* mutations and MSI (microsatellite instability) and TMB (tumor mutational burden) status.

### 2.2. Treatment, Response Assessment and Follow-Up

Patients received investigational therapies per individual trial protocols. For those treated with immunomodulatory agents, therapy was administered according to protocol-defined schedules and continued until radiographic or clinical disease progression, unacceptable toxicity, withdrawal of consent, or death.

Baseline radiographic evaluation was performed prior to trial initiation using contrast-enhanced computed tomography (CT). Tumor assessments were subsequently performed every 8–12 weeks (approximately every 3–4 treatment cycles) or earlier if clinically indicated. Radiographic response was evaluated using Response Evaluation Criteria in Solid Tumors (RECIST), version 1.1.

Progression-free survival (PFS) was defined as the time from early-phase trial enrollment to documented disease progression or death from any cause, whichever occurred first. Overall survival (OS) was defined as the time from trial enrollment to death from any cause or last follow-up. Patients without an event were censored at the date of last clinical contact.

Following treatment discontinuation, patients were monitored at approximately 2- to 3-month intervals.

*KRAS*, *TP53*, *SMAD4*, *CDKN2A* mutation and MSI, TMB status were determined using next-generation sequencing (NGS) performed on tumor tissue and/or circulating tumor DNA (ctDNA) from plasma samples, as available.

### 2.3. Statistical Analysis

Baseline patient characteristics are summarized using descriptive statistics. Categorical variables are reported as frequencies and percentages, and continuous variables are summarized using medians and ranges. Comparisons between groups were performed using the chi-square or Fisher’s exact test for categorical variables and Wilcoxon rank-sum test for continuous variables, as appropriate.

Survival outcomes were estimated using the Kaplan–Meier (KM) method. To account for the non-proportional hazards observed in the crossing KM curves, the restricted mean survival time (RMST), the average survival time from time 0 to a pre-specified truncation time (*τ*), was calculated and compared between groups. Truncation times were selected based on the follow-up distribution to include at least 90% of the observed events and were set at τ=24 months for OS and τ=20 months for PFS.

Exploratory subgroup analyses were performed to evaluate the association between immunomodulatory therapy exposure and survival for each KRAS subtype group. Fleming–Harrington weighted log-rank tests were used to evaluate differences in survival distributions under potential non-proportional hazards, with weighting schemes selected to emphasize the timing of separation observed in the KM curves. Specifically, FH (1,0), FH (1,1), and FH (0,1) weights were used to emphasize early, intermediate, and late differences, respectively. The standard log-rank test, corresponding to FH (0,0), is also reported when appropriate. Selection of Fleming–Harrington weighting schemes was performed post hoc based on observed patterns of survival curve separation and should be considered exploratory. Univariate and multivariable Cox proportional hazard regression models were used to evaluate associations between clinical and molecular variables and survival outcomes. Hazard ratios (HRs) with 95% confidence intervals (CIs) are reported. Variables included in multivariable Cox models were selected based on clinical relevance and potential confounding, including age, sex, pancreatic resection, number of prior systemic therapy lines, liver metastases at trial entry, *KRAS* status, *TP53* status, prior radiation therapy, and immunomodulatory therapy exposure. The proportional hazard assumption was assessed using Schoenfeld residuals. For the multivariable Cox model for OS, pancreatic resection status was used as a stratification factor to address evidence of non-proportional hazards, allowing baseline hazards to differ by resection status. In the multivariable PFS model, no evidence of violation of the proportional hazard assumption was observed. Therefore, pancreatic resection was retained as a standard covariate for the PFS.

Immunomodulatory therapy exposure was primarily analyzed as a binary variable (yes vs. no). In an exploratory post hoc analysis, survival outcomes were additionally compared between patients receiving checkpoint inhibitors and patients receiving non-checkpoint immunomodulatory therapies using the KM method and log-rank tests. *KRAS* mutation status and *KRAS* subtype were analyzed as categorical variables using prespecified reference groups. Subgroup analyses assessing the association between immunotherapy exposure and survival were performed within *KRAS*-mutant tumors and according to individual *KRAS* subtypes. Given the exploratory nature of these subgroup analyses and limited sample sizes, no adjustment for multiple comparisons was performed. Primary analyses comparing *KRAS*-mutant and *KRAS* wild-type tumors were restricted to patients with known *KRAS* status. Patients with unknown *KRAS* status were excluded from *KRAS*-stratified analyses but included in overall descriptive summaries where appropriate.

All statistics tests were two-sided, and *p*-value < 0.05 was considered statistically significant. Statistical analyses were performed using R (version 4.5.1).

## 3. Results

### 3.1. Patient Characteristics

A total of 109 patients with advanced PDAC treated in early-phase clinical trials between August 2014 and August 2023 were included in the analysis. The median age was 65 years, and 83% of patients had liver metastases at the time of trial enrollment.

**Figure 1:**
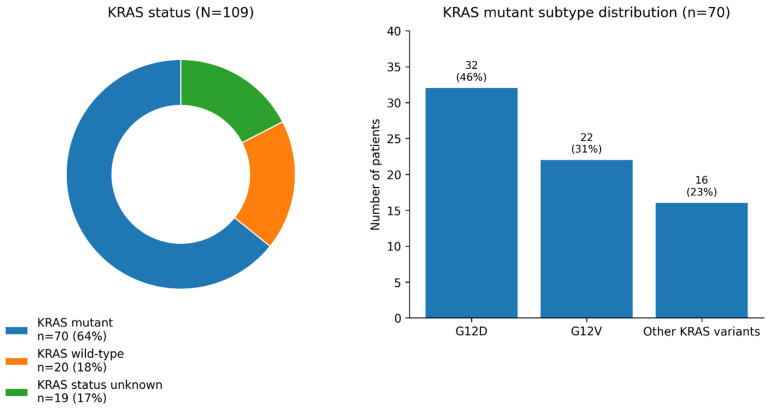
KRAS mutation status and subtype distribution. Among 109 patients with advanced pancreatic ductal adenocarcinoma, 70 (64%) had KRAS-mutant tumors, 20 (18%) were KRAS wild type, and 19 (17%) had unknown KRAS status. Among KRAS-mutant tumors (n = 70), KRAS G12D (n = 32, 46%) and G12V (n = 22, 31%) were most common, with other variants accounting for 16 cases (23%).

*KRAS* mutation status was available in 90 patients (83%). Of the total cohort, 70 patients (64%) harbored *KRAS* mutations, 20 patients (18%) were *KRAS* wild type, and 19 patients (17%) had unknown *KRAS* status (Figure 1). Of 34 patients that received immunomodulator therapy, 12 received checkpoint inhibitors, and others received different immunomodulator therapies that had different mechanisms of action (Table 1). The distribution of checkpoint inhibitor and non-checkpoint immunomodulatory therapies did not differ significantly according to *KRAS* mutation status (*p* = 0.4).

Among *KRAS*-mutant tumors, subtype distribution was as follows: G12D (46%), G12V (31%), G12R (13%), and other *KRAS* variants (10%). Immunomodulatory agents were administered to 39% of patients overall and were most frequently used in the first-line setting (49%). Baseline demographic and clinical characteristics are summarized per *KRAS* status in Table 2 and per immunotherapy exposure in Table 3.

### 3.2. Survival Outcomes in the Overall Cohort

For the entire cohort, the median OS was 5.65 months, and the median PFS was 2.73 months. Restricted mean OS and PFS did not differ significantly between patients with *KRAS*-mutant and *KRAS* wild-type tumors. Over the 24-month follow-up period, the restricted mean OS was 7.54 months in the *KRAS*-mutant group and 8.65 months in the *KRAS* wild-type group (Δ RMST = −1.1 months, 95% CI −4.57 to 2.35; *p* = 0.53). Restricted PFS over 20 months was 3.82 vs. 4.24 months (Δ RMST = −0.46 months, 95% CI −2.55 to 1.72; *p* = 0.7). In the exploratory analysis comparing survival outcomes by immunomodulatory therapy subgroup, checkpoint inhibitor recipients showed a longer median OS compared to non-checkpoint immunomodulatory therapy recipients, although this difference did not reach statistical significance (6.7 vs. 3.25 months, standard log-rank *p* = 0.08). The median PFS was also not significantly different between the immunomodulatory subgroups (median PFS 1.94 vs. 1.58 months, standard log-rank *p* = 0.12).

**Figure 2:**
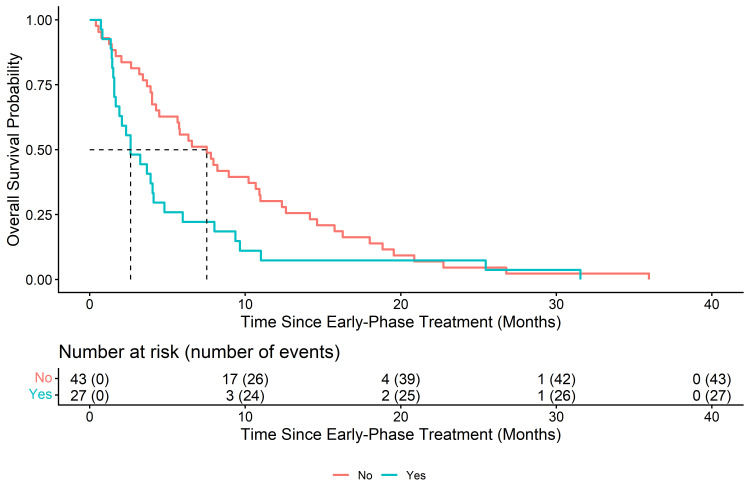
Overall survival by immunomodulatory therapy exposure among patients with KRAS-mutant advanced pancreatic ductal adenocarcinoma. Patients with KRAS-mutant PDAC, receipt of immunomodulatory therapy was associated with significantly shorter OS compared with those who did not receive immunomodulatory therapy (median OS 2.63 vs. 7.52 months, standard log-rank, *p* = 0.03).

Among patients with *KRAS*-mutant PDAC, receipt of immunomodulatory therapy was associated with significantly shorter OS compared with those who did not receive immunomodulatory therapy (median OS 2.63 vs. 7.52 months, standard log-rank, *p* = 0.03) (Figure 2).

**Figure 3:**
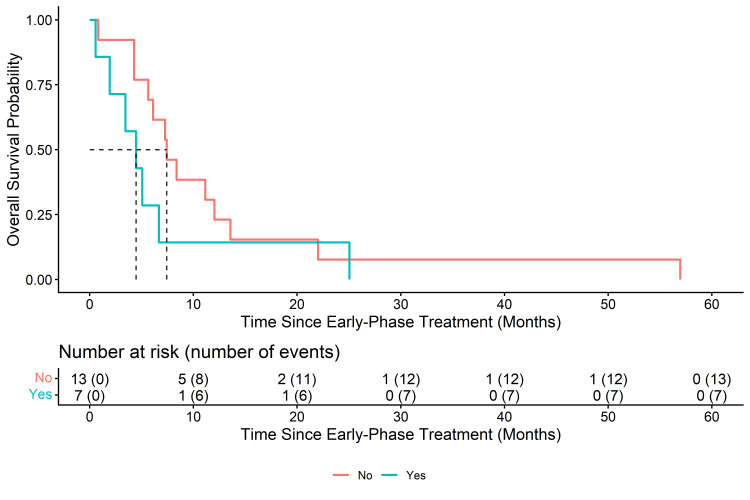
Overall survival by immunomodulatory therapy exposure among patients with KRAS wild-type advanced pancreatic ductal adenocarcinoma. Patients with KRAS wild-type tumors, immunomodulatory therapy exposure was not associated with a statistically significant difference in OS (median OS 4.47 vs. 7.43 months, standard log-rank, *p* = 0.2).

In contrast, among patients with *KRAS* wild-type tumors, immunomodulatory therapy exposure was not associated with a statistically significant difference in OS (median OS 4.47 vs. 7.43 months, standard log-rank, *p* = 0.2) (Figure 3).

### 3.3. Survival Outcomes by KRAS Subtype

**Figure 4:**
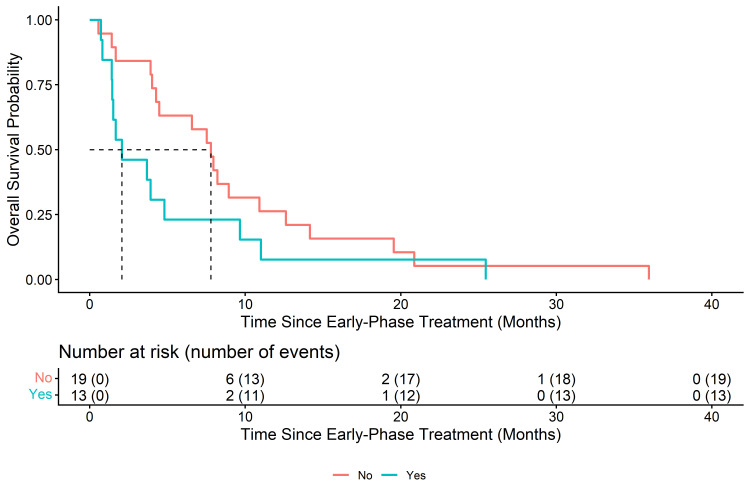
Overall survival by immunomodulatory therapy exposure among patients with KRAS G12D-mutant advanced pancreatic ductal adenocarcinoma. Patients received immunomodulatory therapy had a median OS of 2.07 months compared with 7.79 months in those who did not receive immunomodulatory therapy (Fleming–Harrington FH (1,0) weighted log-rank test, *p* = 0.04).

When stratified by *KRAS* subtype, the association between immunomodulator exposure and inferior OS was most pronounced in patients with *KRAS* G12D-mutant tumors. Among these patients, those who received immunomodulatory therapy had a median OS of 2.07 months compared with 7.79 months in those who did not receive immunomodulatory therapy (Fleming–Harrington FH (1,0) weighted log-rank test, *p* = 0.04) (Figure 4).

In contrast, no statistically significant differences in OS according to immunomodulatory therapy exposure were observed among patients with *KRAS* G12V mutations (median OS 4.04 vs. 10.22 months, Fleming–Harrington FH (1,1) weighted log-rank test, *p* = 0.48) or other KRAS variants (median OS 2.33 vs. 5.75 months, Fleming–Harrington FH (1,1) weighted log-rank test, *p* = 0.20). These findings are summarized in Table 4.

### 3.4. Univariate and Multivariable Analyses

On univariate Cox regression analysis, immunomodulatory therapy exposure, higher number of prior lines of therapy, and presence of liver metastases were each associated with inferior OS.

**Figure 5:**
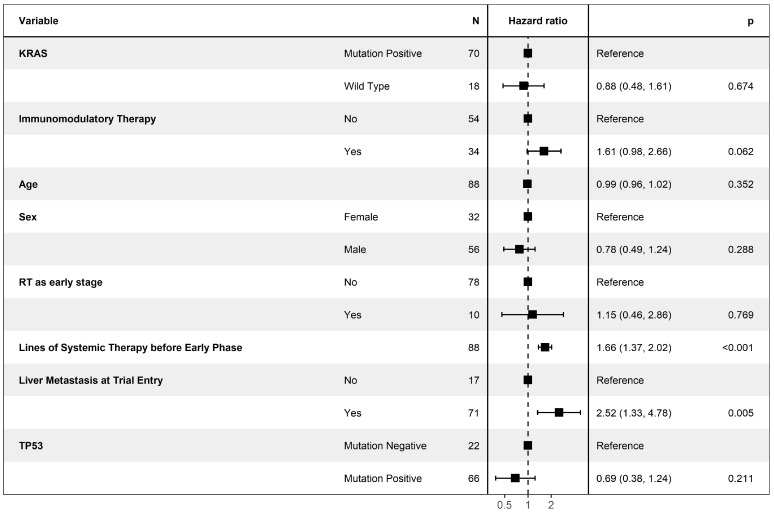
Forest plot for adjusted HR: overall survival.

On multivariable analysis adjusting for relevant clinical covariates, immunomodulatory therapy exposure demonstrated a non-significant trend toward worse OS (hazard ratio [HR] 1.61, 95% confidence interval [Cl] 0.98–2.66; *p* = 0.06) (Table 5, Figure 5).

In contrast, number of prior treatment lines and presence of liver metastases remained independently associated with inferior OS, suggesting that unadjusted association between immunomodulatory therapy exposure and survival was partially confounded by disease burden and prior treatment intensity.

## 4. Discussion

In this exploratory retrospective study, we observed that outcomes with immunomodulatory therapy in advanced PDAC may differ according to *KRAS* mutational subtype, revealing clinically meaningful heterogeneity within *KRAS*-mutant disease. In particular, patients with *KRAS*-mutant tumors—most notably those harboring *KRAS* G12D—experienced inferior survival when treated with immunomodulatory agents in early-phase clinical trials. These findings suggest that *KRAS* subtype represents a biologically relevant determinant of immunotherapy response in PDAC and support biomarker-driven approach for therapeutic selection.

Although immunotherapy has historically demonstrated limited activity in populations with unselected PDAC [23], our data suggests that outcomes may not be uniformly distributed across *KRAS* subtypes.

Our findings are consistent with preclinical models demonstrating that distinct *KRAS* mutational subtypes differentially modulate tumor biology and the immune microenvironment in pancreatic cancer [33,34]. To our knowledge, this study represents the first clinical analysis to demonstrate differential immunomodulatory therapy outcomes according to *KRAS* subtype in pancreatic cancer, providing translational validation of these preclinical observations.

Similar subtype-dependent effects have been described in other malignancies. In lung adenocarcinoma, *KRAS* mutation subtypes have been associated with differential responses to immune checkpoint inhibitors, reflecting distinct tumor immune microenvironments [35,36,37,38]. Notably, *KRAS* subtype has not consistently correlated with PD-L1 expression, suggesting that functional immune contexture rather than biomarker expression underlies therapeutic variability [39]. In colorectal cancer, *KRAS* variants have been associated with differential immune infiltration patterns [40]. Jeong et al. [33] demonstrated that *KRAS* mutation subtypes induced changes in the overall immune system environment in PDAC. Together, these observations support the hypothesis that *KRAS* mutations are not biologically equivalent and differentially shape tumor–immune interactions across tumor types.

The prevalence of *KRAS* mutations in our cohort was lower than that reported in prior pancreatic cancer series [33,41]. This likely reflects the unique referral pattern of an early-phase clinical trial, where patients with more favorable performance status and longer survival are over-represented. Because *KRAS* wild-type PDAC has been associated with distinct molecular features and improved prognosis, this enrichment may partially explain the higher proportion of *KRAS* wild-type tumors observed in our study.

Immunotherapy response in PDAC is likely influenced not only by *KRAS* mutational subtypes but also by co-occurring genomic alterations that shape tumor biology and the immune composition. In our cohort, *TP53* alterations were present in the majority of KRAS-mutant tumors; however, TP53 mutation was not associated with differential immunotherapy outcomes, consistent with prior reports [42]. Although *SMAD4*, *CDKN2A*, MSI, and TMB status were available for a subset of patients, the extent of missing data and limited sample size precluded robust evaluation of their association with immunotherapy outcomes and may represent important modifiers of immune responsiveness. More generally, genomically annotated cohorts incorporating comprehensive co-mutation analysis will be essential to evaluate these interactions.

Another important consideration is the low prevalence of established immunotherapy biomarkers within our cohort. Among patients with available molecular data, MSI-high and TMB-high tumors were rare, consistent with the known biology of PDAC. The predominance of MSI-low and TMB-low tumors likely contributed to the limited efficacy observed within immunomodulatory approaches, particularly for immune checkpoint inhibitors. It is important to note, however, that the majority of immunomodulatory therapies included in this study were investigational agents designed to modulate innate immunity, the tumor microenvironment, or myeloid cell function and were not selected based on MSI or TMB status. Nonetheless, future studies evaluating immunomodulatory therapy strategies in PDAC should incorporate comprehensive biomarker profiling, including MSI and TMB assessment, to better define patient populations most likely to benefit.

From a therapeutic perspective, our findings suggest that *KRAS* subtype informs rational combination strategies integrating *KRAS*-targeted agents with immunomodulatory therapies. Emerging *KRAS* inhibitors have demonstrated subtype-specific activity, and their combination with immunotherapies or other immune modulating agents may enhance antitumor immunity in a context-dependent manner. Different KRAS subtypes may require distinct immunomodulatory partners—such as immune checkpoint inhibitors, myeloid-targeting agents, or innate immune activators—to overcome subtype-specific mechanisms of immune resistance. The recently reported phase III RASolute 302 trial further highlights the clinical relevance of targeting the RAS pathway in pancreatic cancer [43]. In this study, the pan-RAS inhibitor daraxonrasib significantly improved survival outcomes compared with standard therapy in previously treated RAS-mutant PDAC, establishing broad RAS inhibition as a promising therapeutic strategy. Preclinical studies have demonstrated that KRAS inhibition can remodel the tumor microenvironment, enhance antigen presentation, increase CD8+ T-cell infiltration, and partially reverse immune suppression. As effective RAS-targeted therapies become increasingly available, future patient selection for immunomodulatory strategies may incorporate not only KRAS mutational status and subtype but also sensitivity to RAS pathway inhibition. Our findings support the concept that KRAS-subtype-specific biology may remain an important determinant of therapeutic response even in the era of pan-RAS inhibition and should be considered in the design of future biomarker-driven combination studies.

Importantly, in multivariable analysis, immunomodulatory therapy exposure demonstrated only a non-significant trend toward inferior survival, while disease burden and prior treatment intensity remained independently prognostic. This suggests that the observed associations are influenced by other clinical confounders. The substantial imbalance in treatment line between exposure groups warrants particular caution in interpretation. Patients receiving immunomodulatory therapies were considerably more heavily pretreated than those receiving non-immunomodulatory therapies, reflecting referral patterns and trial availability within an early-phase program. Although multivariable adjustment attenuated the association between immunomodulatory therapy exposure and survival, residual confounding remains possible.

This study has several limitations. First, its retrospective design introduces the potential for selection bias and unmeasured confounding. Second, the sample size was modest, particularly within individual KRAS subtype subgroups, limiting statistical power. Third, immunomodulatory therapy exposure encompassed a heterogeneous group of agents with distinct mechanisms of action, which may have diluted subtype-specific treatment effects. Although a post hoc subgroup analysis was conducted to compare survival outcomes between checkpoint inhibitor and non-checkpoint immunomodulatory therapies and no statistically significant differences were observed, these findings were underpowered and should be interpreted as inconclusive rather than as evidence of equivalent efficacy between the subtypes. In addition, further stratification within individual KRAS subtypes was not feasible due to sample size constraints, as each KRAS and immunomodulatory therapy subtype combination only had less than 10 patients, which precludes reliable survival estimation. Further studies with larger cohorts are needed to adequately study the heterogeneity of immunomodulatory agents within KRAS subtypes. Fourth, patients receiving immunomodulatory therapies were more heavily pretreated than those receiving non-immunomodulatory therapies, introducing potential confounding despite multivariable adjustment. Fifth, molecular data for MSI, TMB, SMAD4, and CDKN2A were incomplete in a substantial proportion of patients, limiting our ability to evaluate the influence of these biomarkers on treatment outcomes. Sixth, immune microenvironmental correlates, including tumor-infiltrating lymphocytes, PD-L1 expression, and cytokine profiling, were not available. Finally, no adjustment for multiple comparisons was performed in the exploratory subgroup analyses, and the findings should therefore be considered hypothesis-generating.

Despite these limitations, our findings provide clinical rationale supporting *KRAS*-subtype-specific heterogeneity in immunotherapy outcomes in PDAC. These results warrant prospective validation and suggest that future clinical trials should incorporate *KRAS* mutational subtype stratification and consider immunomodulatory agent class as a stratification or randomization factor when evaluating immunomodulatory strategies in pancreatic cancer.

## 5. Conclusions

In this retrospective analysis of patients with advanced PDAC enrolled in early-phase clinical trials, KRAS mutation status alone was not associated with significant differences in progression-free or overall survival. However, exploratory analyses suggested that outcomes vary according to specific KRAS subtype, particularly among patients treated with immunomodulatory therapies. These findings support the growing recognition that KRAS-mutant pancreatic cancer is a biologically heterogeneous disease and that individual KRAS subtypes influence interactions with the tumor immune microenvironment.

Although limited by the retrospective design, modest sample size, and heterogeneous treatment approaches, this study provides hypothesis-generating evidence that KRAS subtype represents a clinically relevant factor for patient stratification in future therapeutic studies. Prospective validation in larger cohorts is warranted to clarify the predictive and prognostic significance of KRAS subtypes and to guide the development of more personalized treatment strategies for pancreatic cancer.

## Figures and Tables

**Table 1:** Distribution and mechanistic classification of immunomodulatory agents by KRAS status.

	Agent	Mechanism of Action	KRAS Wild Type (n = 20)	KRAS Mutant (n = 70)	Overall (n = 90)
Checkpoint inhibitors		Immune checkpoint blockade	1 (5%)	11 (15.8%)	12 (13.3%)
Non-checkpoint inhibitors	BNT411	Innate Immune activator	1 (5%)	4 (5.7%)	5 (5.6%)
	IMSA101	Innate immune activator	0 (0%)	3 (4.3%)	3 (3.3%)
	Q702	Myeloid/TME modulator	1 (5%)	1 (1.4%)	2 (2.2%)
	VT1021	Myeloid/TME modulator	4 (20%)	8 (11.4%)	12 (13.3%)
No immunomodulator therapy			13 (65%)	43 (61.4%)	56 (62.3%)

Footnote: Among patients with KRAS mutations receiving immunomodulatory therapy, the distribution of agents by KRAS subtype was as follows: KRAS G12D (n = 13): checkpoint inhibitor (n = 3), BNT411 (n = 4), IMSA101 (n = 1), Q702 (n = 1), and VT1021 (n = 4); KRAS G12V (n = 7): checkpoint inhibitor (n = 4), IMSA101 (n = 1), and VT1021 (n = 2); other KRAS variants (n = 7): checkpoint inhibitor (n = 4), IMSA101 (n = 1), and VT1021 (n = 2). These data are presented descriptively because of the limited sample size within each subgroup.

**Table 2:** Baseline patient characteristics by KRAS mutation status.

Characteristics	KRAS Wild Type (n = 20)	KRAS Mutant (n = 70)	*p*-Value	Overall (n = 90)
Age, median (Q1–Q3), years	72 (62–74)	65 (58–69)	0.10	65 (58–71)
Gender, n (%)			0.5	
Female	6 (30)	27 (39)		33 (37)
Male	14 (70)	43 (61)		57 (63)
Radiotherapy as early-stage treatment, n (%)			>0.9	
No	18 (90)	62 (89)		80 (89)
Yes	2 (10)	8 (11)		10 (11)
Pancreatic resection, n (%)			>0.9	
No	14 (78)	55 (79)		69 (78)
Yes	4 (22)	15 (21)		19 (22)
unknown	2	0		2
Lines of systemic therapy prior to early-phase trial, n (%)			0.9	
0	10 (50)	31 (44)		41 (46)
1	2 (10)	9 (13)		11 (12)
2	5 (25)	21 (30)		26 (29)
3	2 (10)	7 (10)		9 (10)
4	1 (5)	1 (1.4)		2 (2.2)
≥5	0 (0)	1 (1.4)		1 (1.1)
Reason for discontinuation of early-phase trial, n (%)			0.3	
Progression	11 (55)	47 (67)		58 (64)
Side effects	2 (10)	2 (2.9)		4 (4.4)
Death	7 (35)	21 (30)		28 (31)
Liver metastases at trial entry, n (%)			0.5	
No	5 (25)	12 (17)		17 (19)
Yes	15 (75)	58 (83)		73 (81)
Immunomodulatory treatment received on trial, n (%)			0.8	
No	13 (65)	43 (61)		56 (62)
Yes	7 (35)	27 (39)		34 (38)
TP53 status, n (%)			***<0.001***	
Mutation-negative	11 (55)	12 (17)		23 (21)
Mutation-positive	9 (45)	58 (83)		67 (61)
SMAD4 status, n (%)			***0.009***	
Mutation-negative	8 (40)	45 (64)		53 (59)
Mutation-positive	0 (0)	8 (11)		8 (8.9)
Unknown	12 (60)	17 (24)		29 (32)
CDKN2A status, n (%)			***0.008***	
Mutation-negative	7 (35)	34 (49)		41 (46)
Mutation-positive	1 (5)	19 (27)		20 (22)
Unknown	12 (60)	17 (24)		29 (32)
MSI status, n (%)			***<0.001***	
Low	6 (30)	50 (71)		56 (62)
Unknown	14 (70)	20 (29)		34 (38)
TMB status, n (%)			***<0.001***	
High	1 (5)	4 (5.7)		5 (5.6)
Low	4 (20)	47 (67)		51 (57)
Unknown	15 (75)	19 (27)		34 (38)

*p*-values that reached statistical significancve (*p* < 0.05) are shownin bold and italics.

**Table 3:** Baseline patient characteristics by immunomodulator therapy exposure.

Characteristics	No Immunomodulator Therapy Exposure (n = 56)	Yes Immunomodulator Therapy Exposure (n = 34)	*p*-Value	Overall (n = 90)
Age, median (Q1–Q3), years	65 (58–72)	66 (59–71)	>0.9	65 (58–71)
Gender, n (%)			0.8	
Female	21 (38)	12 (35)		33 (37)
Male	35 (63)	22 (65)		57 (63)
Radiotherapy as early-stage treatment, n (%)			0.5	
No	51 (91)	29 (85)		80 (89)
Yes	5 (8.9)	5 (15)		10 (11)
Pancreatic resection, n (%)			0.7	
No	43 (80)	26 (76)		69 (78)
Yes	11 (20)	8 (24)		19 (22)
Unknown	2	0		2
Lines of systemic therapy prior to early-phase trial, n (%)			***<0.001***	
0	39 (70)	2 (5.9)		41 (46)
1	2 (3.6)	9 (26)		11 (12)
2	8 (14)	18 (53)		26 (29)
3	4 (7.1)	5 (15)		9 (10)
4	2 (3.6)	0 (0)		2 (2.2)
≥5	1 (1.8)	0 (0)		1 (1.1)
Reason for discontinuation of early-phase trial, n (%)			0.057	
Progression	39 (70)	19 (56)		58 (64)
Side effects	4 (7.1)	0 (0)		4 (4.4)
Death	13 (23)	15 (44)		28 (31)
Liver metastases at trial entry, n (%)			0.7	
No	10 (18)	7 (21)		17 (19)
Yes	46 (82)	27 (79)		73 (81)
KRAS status, n (%)			0.8	
Wild type	13 (23)	7 (21)		20 (22)
Mutation-positive	43 (77)	27 (79)		70 (78)
TP53 status, n (%)			0.2	
Mutation-negative	12 (21)	11 (32)		23 (26)
Mutation-positive	44 (79)	23 (68)		67 (74)

*p*-values that reached statistical significancve (*p* < 0.05) are shown in bold and italics.

**Table 4:** Overall survival and progression-free survival by immunotherapy exposure according to *KRAS*-mutant subtype.

*KRAS* Subtype	Immunotherapy Exposure	n	Median OS (95% CI) Months	*p*	Median PFS (95% CI) Months	*p*
**G12D**	**Yes**	**13**	**2.07 (1.45–NE)**	***0.04***	**1.48 (1.18–NE)**	**0.09**
	**No**	**19**	**7.79 (4.47–14.2)**		**2.96 (1.71–7.29)**	
**G12V**	**Yes**	**7**	**4.04 (2.63–NE)**	**0.5**	**1.71 (1.38–NE)**	**0.3**
	**No**	**15**	**10.22 (3.68–16.3)**		**3.35 (1.54–5.72)**	
**Other** ***KRAS*** **mutations**	**Yes**	**7**	**2.33 (1.58–NE)**	**0.14**	**1.91 (1.58–NE)**	**0.3**
	**No**	**9**	**5.75 (2.66–NE)**		**2.66 (1.81–NE)**	

OS, overall survival; PFS, progression-free survival. NE: not estimable because insufficient events/limited follow-up means the survival CI band does not cross 50%, so there are no data supporting upper bound. *p*-values that reached statistical significancve (*p* < 0.05) are shownin bold and italics.

**Table 5:** Univariate and multivariable cox regression analyses for overall survival (OS) and progression-free survival (PFS).

Variable	OS– Univariate HR (95% Cl)	*p*	OS–Multivariable HR (95% Cl)	*p*	PFS– Univariate HR (95% Cl)	*p*	PFS– Multivariable HR (95% Cl)	*p*
**Age**	**0.984 (0.959–1.010)**	**0.2**	**0.986 (0.957–1.016)**	**0.4**	**0.989 (0.963–1.015)**	**0.4**	**0.995 (0.967–1.023)**	**0.7**
**Gender** **Female** **Male**	**0.718 (0.460–1.118)**	**0.14**	**0.776 (0.486–1.239)**	**0.3**	**0.758 (0.490–1.173)**	**0.2**	**0.706 (0.435–1.145)**	**0.2**
**Radiotherapy as early-stage treatment,** **no** **Yes**	**1.122 (0.579–2.174)**	**0.7**	**1.147 (0.459–2.864)**	**0.8**	**0.811 (0.417–1.577)**	**0.5**	**0.522 (0.211–1.293)**	**0.2**
**Lines of systemic therapy prior to early-phase trial**	**1.553 (1.315–1.836)**	***<0.001***	**1.660 (1.366–2.018)**	***<0.001***	**1.332 (1.130–1.571)**	***<0.001***	**1.258 (1.055–1.500)**	***0.01***
**Liver metastases at trial entry, n (%)** **No** **Yes**	**1.817 (1.009–3.273)**	***0.047***	**2.522 (1.331–4.779)**	***0.005***	**0.906 (0.533–1.541)**	**0.7**	**0.896 (0.504–1.596)**	**0.7**
**Immunomodulatory treatment received during trial,** **No** **Yes**	**1.779 (1.149–2.754)**	***0.010***	**1.613 (0.977–2.663)**	**0.062**	**1.566 (1.014–2.416)**	***0.043***	**1.489 (0.900–2.465)**	**0.12**
**KRAS,** **Mutation positive** **wild type**	**0.797 (0.479–1.326)**	**0.4**	**0.877 (0.477–1.613)**	**0.7**	**0.826 (0.497–1.375)**	**0.5**	**0.969 (0.519–1.807)**	**>0.9**
**TP53,** **Mutation-negative** **Mutation-positive**	**0.784 (0.486–1.264)**	**0.3**	**0.687 (0.382–1.237)**	**0.2**	**1.032 (0.641–1.662)**	**0.9**	**1.154 (0.641–2.081)**	**0.6**
**Pancreatic resection**	**0.944 (0.563, 1.584)**	**0.8**			**0.688 (0.395, 1.197)**	**0.2**	**0.897 (0.444, 1.813)**	**0.8**

*p*-values that reached statistical significancve (*p* < 0.05) are shownin bold and italics.

## Data Availability

The data presented in this study are available on request from the corresponding author.
